# Mindfulness Practice and Job Performance in Social Workers: Mediation Effect of Work Engagement

**DOI:** 10.3390/ijerph191710739

**Published:** 2022-08-29

**Authors:** Chien-Chung Huang, Bin Tu, Huiyu Zhang, Jamie Huang

**Affiliations:** 1 School of Social Work, Rutgers University, New Brunswick, NJ 08901, USA; 2 Guangdong Research Center for NPO, Guangdong University of Foreign Studies, Guangzhou 510420, China; 3 School of Public Administration, Guangdong University of Foreign Studies, Guangzhou 510420, China; 4 Huamin Research Center, Rutgers University, New Brunswick, NJ 08901, USA

**Keywords:** job demands, job performance, mindfulness practice, social workers, work engagement

## Abstract

Despite a rapid increase in the work force over the last decade, the social work labor force is still suffering through high amounts of stress and burnout that could negatively affect work engagement and job performance in China. A potential solution worth exploring, however, is the practice of mindfulness, a concept based on expanding one’s awareness to target focus without judgement. Using 537 social workers from street-level social work service stations in Guangzhou, China, this paper examines the relation between mindfulness practice and job performance, and whether work engagement mediated the relation through the application of the job demand and resources theory. The findings indicate that that mindfulness practice directly increases work engagement (Beta = 0.33) and has an indirect effect on job performance (Beta = 0.21) through its effect on work engagement that fully mediated the relation between mindfulness practice and job performance. In contrast to formal mindful practices (Beta = 0.13), informal mindful practices (Beta = 0.22) encompass a broader impact on employee performance. The findings suggest that mindfulness practice can effectively be used in workspaces to enhance engagement and performance of social workers in China.

## 1. Introduction

The economy in China has been rapidly evolving since the 1980s. The development is coupled with growing social problems and issues. Consequently, China has seen noteworthy developments to its social work education programs and personnel to respond to increasing social issues. Like Western countries, social work is a practice-based profession that promotes social development and cohesion of people and communities [1,2,3]. Social workers are working with families and institutions to provide services and advance well-being of vulnerable individuals and families [1,2,3,4]. The number of students graduated from social work programs has risen from less than 100 in 1989 to over 40,000 in 2018 [2,3]. Likewise, the number of social workers has increased from 0.2 million in 2010 to 1.5 million in 2020 [2,3,4]. The growth of these statistics highlights the speed of social work expansion in China in the past decades.

Social workers, like other human service employees, encounter considerable burnout and turnover rates across the globe [5,6,7,8,9]. Not surprisingly, Chinese social workers face similar challenges [10,11,12,13]. As studies have shown that burnout and intentions to leave the organization have negative effects on work engagement and job performance [9,13,14,15], the examination of work involvement and job fulfillment is crucial to identifying potential factors that can promote the improvement of engagement and performance. In hopes of shedding light on the effects of mindfulness practice on work engagement and job performance amongst social workers in China, this research study applies the Job Demands and Resources theory and strives to inspect how mindfulness practice, a resource, affects job performance and whether work engagement mediates the relationship between mindfulness and job performance in social workers in China, an evolving profession with high job demands.

### 1.1. Work Engagement and Job Performance

Work engagement is a fulfilling, work-related, and positive state of mind exemplified by the strength of dedication and vigor [16,17]. Job performance is the efficiency with which an employee performs the work that contributes to an organization’s overall success and comprises two main factors: task and contextual performances [18,19]. Task performance is the core job responsibility of an employee or in-role prescribed behavior, reflected in delivering specific work outcomes and deliverables. Contextual performance refers to the discretionary extra-role behavior beyond formal job responsibilities such as coaching coworkers. Contextual performance has been regarded as a vital part of overall job performance [20,21,22].

There is evidence proving that work engagement leads to high job performance, including both task and contextual performances [23,24,25]. For example, Meyers et al. [21] used 753 Dutch employees and showed that work engagement was positively related to contextual performance. Halbesleben and Wheeler [23] found that of 587 employees from various industries and occupations, work engagement was related to high task performance. Additionally, Song and colleagues [26] also found that amongst a sample of 481 Korean teachers, work engagement was positively correlated with task performance.

### 1.2. Mindfulness and Mindfulness Practice

Mindfulness is a state of consciousness which an individual engages in focused awareness to the current moment and maintains non-judgmental reactions to the surrounding [27,28]. Empirical research on mindfulness has shown that it has positive effects on health and well-being [26,29,30,31]. Studies also indicated that high level of mindfulness was linked to less exhaustion and positively interconnected with well-being at work [32,33]. Indeed, mindfulness acts as a personal resource and can boost productivity while setting a radiant mood for an individual [34,35,36,37]. Mindfulness can be applied to assist an individual in remaining a tranquil and object demeanor when faced with stressful situations that provoke emotional responses. Alternatively, it can aid an individual to remain present and alert of the instant while evading possible interruptions at the workspace. Accordingly, workers can apply mindfulness to increase attention and positive affect, reduce negative affect and burnout, and improve work engagement and job performance [38,39,40,41]. Empirical evidence has shown that mindfulness practice can lessen negative affect and advance positive affect and authentic functioning, both of which were associated with ameliorating work engagement and job performance [34,35,42,43,44].

Mindfulness practice can be conducted formally or informally. Formal mindfulness practice, such as meditation, is a set of methods that are planned to boost a keen state of awareness [45,46]. Informal mindfulness practice entails the use of an aware perception in everyday doings, such as walking, eating, exercising, or dishwashing [47,48,49]. Empirical research has shown that both formal and informal mindfulness practices were related to positive health and well-being [47,48,49,50].

## 2. Conceptual Framework and Hypothesis

The Job Demands and Resources (JD-R) theory speculates that job demands and resources influence work engagement and performance through two processes: the health-impairment and the motivation-driven processes [51,52]. Job demands refer to the various work conditions that need a constant physical or mental effort. Frequently, this constant effort inflicts a physiological cost on the worker, such as exhaustion, leading to a gradual depletion of their energy. Through these health-impairing processes, job demands will impact efficacy. In contrast, job resources are parts of the job that can accelerate the accomplishment of a task, alleviate the psychological cost of job demands, and improve work engagement and performance [51,52]. Despite the lack of mindfulness practice encouragement from social work agencies, workers can still utilize these exercises as a personal resource to stimulate enhanced positive affect and lessen negative affect within workspaces, allowing for elevated labor engagement and performance [22,26,35,42,43].

**Figure 1:**
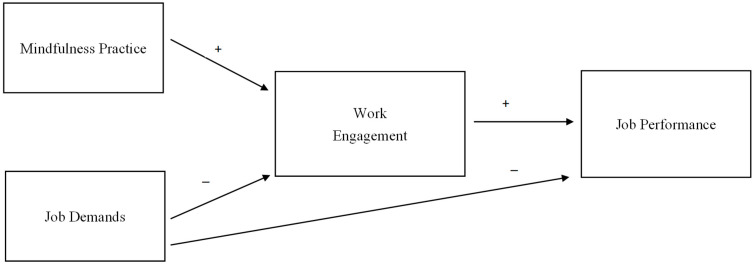
Conceptual model.

The widely tested JD-R theory that is backed by numerous studies and have affirmed its validity in examining employment stress and burnout, health, work engagement, and job performance [53,54,55,56,57]. Yet, studies on practicing mindfulness, work engagement, and job performance in social workers are limited in China. We, thus, utilize the JD-R theory and propose that mindfulness practice as a resource that can help social workers’ work engagement which has positive effects on job performance, while job demands affect work engagement and job performance via the health-impairment process. That is, we hypothesize a mediation model that work engagement mediates the relation between mindfulness practice and job performance. The conceptual model of this study is illustrated in Figure 1. Specifically, we test the subsequent hypotheses amongst a sample of Chinese social workers:

**Hypothesis** **1.** *Practicing mindfulness is positively associated with work engagement.*

**Hypothesis** **2.** *JD are negatively associated with work engagement.*

**Hypothesis** **3.** *JD are negatively associated with job performance.*

**Hypothesis** **4.** *Work engagement mediates the relation between mindfulness practice and job performance.*

## 3. Methods

### 3.1. Data

We used an online and anonymous survey to collect the sample for this study. We targeted our sample to one city, Guangzhou, China, as it shows a swift expansion in social work in that city [58]. The Guangzhou government created social work service stations at the street-level in 2017, where the government purchased social work services from registered social work agencies to offer accessible social services to the communities in Guangzhou. The service stations provide convenient locations for social workers to provide services to individuals and families in need, including child and adolescent services, health care, women development, family service, elderly care, disability assistance, and community development [58]. There are 180 social work service stations in Guangzhou, and each station was equipped with 20 social workers, with 14 of them front-line social workers. A total of 54 stations were randomly selected for the study. We distributed the survey link to front-line social workers in the selected stations on 15 September 2021. Reminders were sent 7 and 14 days after the initial distribution. Due to the setup in the online survey, there were incomplete surveys, including missing data, and others were not able to finalize the submission of the survey, resulting in missing cases. A total of 537 social workers fully completed the online survey by 10 October 2021, presenting a 71% of response rate (537/[54 × 14 = 756]). Most of the sample were female (84.5%) and never married (54.2%). The average age of the sample was 29-year-olds, and a majority of them had at least a college degree. Informed consent was notified, and participants were informed that their participation was voluntary, and they can end the survey at any time. The research protocol was approved by a review committee at one of the co-author’s universities in China.

### 3.2. Measures

Job performance was assessed in two dimensions: task and contextual performance. The 9-item task performance scale was utilized to assess task performance, while contextual performance was judged by the 7-item contextual performance scale [59]. Multiple studies have verified this scale’s validity, psychometric soundness, and reliability [60,61]. Exampled questions include whether the worker feels they are able to “achieve the objectives of the job” (task performance) and “helps other employees with their work when they have been absent” (contextual performance). Each item was appraised on a seven-point Likert scale (0–6), high scores representing that the item was entirely characteristic of the employee, while a low score meant it was not typical of the employee. We calculated the score of job performance by averaging all items. The Cronbach’s alpha was 0.94 for task performance, 0.93 for contextual performance, and 0.95 for overall job performance in this study.

Work engagement was assessed by the short form of the Utrecht Work Engagement Scale (UWES-9) [62]. Numerous studies have verified the short-form version of this scale for its validity, reliability, and soundness [62,63,64]. The UWES-9 includes 9 items to gauge the vigor, dedication, and absorption dimensions of work engagement. Each has 3 items. Participants were first tasked with answering opinion questions about their jobs. Example questions were: “At my work, I feel bursting with energy” (vigor), “I am enthusiastic about my job” (dedication), and “I am immersed in my work” (absorption). Each item was assessed along a 7-point Likert scale ranging from 0 (“never”) to 6 (“always”). The overall work engagement score was an average score of all 9 items. The Cronbach’s alpha of work engagement was 0.94 in this study.

Mindfulness practice was assessed by inquiring subjects whether they partaken in the subsequent activities: mindful nature observation and meditation. Meditation was used to measure the level of formal practice and mindful nature observation was utilized to gauge informal practice. Each activity was evaluated on a 7-point Likert scale, from 0 (never) to 6 (once or more per day).

Job demands were assessed by the Lequeurre et al.’s Questionnaire sur les Ressources et Contraintes Professionnelles (QRCP) [65], which showed good reliability in the Chinese context [66]. Three dimensions of JD were assessed, including pace and amount of workload, emotional workload, and changes in the tasks. Pace and amount of workload denotes the experience of having excess work tasks within a limited time frame, while emotional workload refers to the emotional energy workers are forced to expel to accomplish certain job demands. Changes in the tasks indicate the difficulties posed to workers by changes in job roles. Four questions were used to measure each dimension. Each item was evaluated using a 7-point Likert scale ranging from 1 (never) to 7 (always). We calculated the JD score by averaging all items. Higher score indicates higher job demands. The Cronbach’s alpha was 0.83 for job demands. Appendix A lists all scale items used in this study.

### 3.3. Analytical Approach

The descriptive analysis for all variables was first performed, followed by correlation analyses. Then, Structural Equation Modeling (SEM) was applied to examine the direct and indirect effects of mindfulness practice on job performance through the hypothesized mediator, work engagement, while controlling for job demands. Alternatively, the regression analyses with extensive covariates, including personal features, were conducted. The results from the regression analyses were similar to those reported here. Results of regression analyses are not provided within this study but can be provided upon request. SEM was preferred because it allows for the simultaneous examination of direct and indirect effects through the mediating variable. The maximum likelihood (ML) estimation was used in SEM. The model-to-data fit was assessed by several fit indices, including Chi-square statistics, Comparative Fit Index (CFI), Root Mean Square Error of Approximation (RMSEA), and Standardized Root Mean Square Residual (SRMR). Values of Chi-square statistics > 0.0.05, CFI > 0.95, RMSEA values < 0.08, and SRMR < 0.08 indicate good model-to-data fit. The normality tests of the variables were examined. The skewness values ranged from −0.36 (work engagement) to 0.16 (job demands) while the kurtosis values were between 2.81 (work engagement) and 3.28 (job performance). The common method variance analysis was performed, and the results showed that only 34% of the variance was shared by mindfulness practice, job demands, work engagement, and job performance items, suggesting that the common method variance was not an issue in the data. The STATA software 16.0 (StataCorp LLC., College Station, TX, USA) was used for all analyses.

## 4. Results

Table 1 reveals the descriptive statistics and correlations of the variables. Respondents had mean mindfulness practice, work engagement, and job performance scores of 2.8, 3.5 and 4.0, respectively. Respondents reported relatively high JD (M = 4.7). The descriptive results suggests that social workers in China had relatively low mindfulness practice, while their work engagement and job performance were above the mean, despite experiencing relatively high job demands.

The findings from correlation analyses were largely consistent with our hypotheses. Mindfulness practice was positively correlated with work engagement (r = 0.34, *p* < 0.001) and job performance (r = 0.22, *p* < 0.001). The findings confirm Hypothesis 1. Consistent with Hypothesis 2, job demands were negative related with work engagement (r = −0.17, *p* < 0.001). Nevertheless, we did not discover a statistical correlation between job demands and job performance (Hypothesis 3). In addition, we find a significant correlation between work engagement and job performance (r = 0.59, *p* < 0.001). There was no correlation between job demands and mindfulness practice.

**Figure 2:**
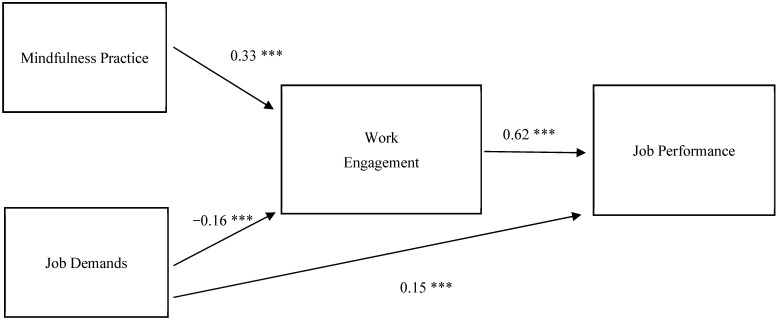
Standardized Estimates of the Hypothesized Model. Note: N = 897; *** *p* < 0.001.

Figure 2 lists the standardized coefficients of the SEM model. The model fit statistics displayed that the proposed model fits adequately to the data: χ^2^ (1) = 0.12, *p* > 0.05, CFI = 1.00, RMSEA = 0.00, SRMR = 0.01. The results show that mindfulness practice was positively associated with work engagement (β = 0.33, *p* < 0.001) while job demands were negatively associated with work engagement (β = −0.16, *p* < 0.001). In conflict with Hypothesis 3, we found job demands had a positive and direct effect on job performance, though the estimate was small (β = 0.15, *p* < 0.01). Work engagement had a significant effect on job performance (β = 0.62, *p* < 0.001). The specification 1 of Table 2 lists indirect and total effects of the SEM model. The indirect effect of mindfulness practice on job performance via work engagement was 0.21 (*p* < 0.001). The above results confirm Hypothesis 4 and indicate that work engagement fully mediated the association between mindfulness practice and job performance. Though job demands have a positive and direct effect on job performance, their indirect and negative effect on job performance via work engagement made the total effect of job demands on job performance insignificant.

We further studied the effects of mindfulness practice on job performance by formal and informal practice. The full estimates were listed in specifications 2 and 3 of Table 2. Informal mindfulness practice had relatively large effects on work engagement (β = 0.36, *p* < 0.01) and job performance (β = 0.22, *p* < 0.01) compared to the ones found in formal practice (β = 0.21 and 0.13, respectively). The results for other variables were similar to the ones presented in Figure 2.

## 5. Discussion

The findings from the SEM supported the hypotheses that mindfulness practice and job demands affect work engagement and job performance among Chinese social workers. Mindfulness practice serves as a resource to advance job performance through increasing work engagement. In contrast, high job demands affect work engagement through the health-impairment process exhibited in the JD-R theory. The estimates of mindfulness practice on work engagement and job performance suggest that social workers can use mindfulness practice to advance their work engagement and have high job performance even when job demands are high. These results are in line with the literature showing that mindfulness has positive effects on work engagement and job performance [34,35,36,37,42,43,44].

The estimate of informal mindfulness practice was larger than that found in formal mindfulness practice. As informal mindfulness practice needs less structure, is easier to execute, and originates the quickest benefit, social workers may benefit from this practice. In addition, informal mindfulness practice was related to the observing facet of mindfulness [67]. This capacity to pay attention to an experience is a crucial mindfulness skill that may stimulate enhanced work engagement and job performance at the workplace. In contrast, workers who engage in formal mindfulness practice might be more conscious of their work engagement and job performance that might affect their self-reports to display low levels of engagement and performance. Further study is warranted to understand the discrepancy between formal and informal practices [48].

The results have practical implications for social work administrators. In contradiction with the JD-R Theory, high job demands in social workers were directly and positively associated with job performance, although the size of the estimate was small (β = 0.15). It may be that social workers are more likely to increase their job performance when facing high job demands because the demands are related to helping vulnerable populations, which make the work meaningful [68]. High job demands, however, have negative effect on work engagement (β = −0.16) which has positive effect on job performance (β = 0.62). As a result, the total effect of job demands on job performance was small and not significant (β = 0.05). The finding was consistent with the correlation results that show no correlation between job demands and job performance. As social workers reported high job demands and a negative association between job demands and work engagement, it is vital that social work administrators pay attention to high job demands and engagement of their workers. To increase work engagement amongst workers, social work administrators might adopt supportive services and caring environments for their workers. In addition, given the findings of mindfulness practice on work engagement and job performance found in this study, along with the literature connection between mindfulness and mental health well-being [32,33], employers may consider introducing mindfulness intervention to their social workers and encourage them to practice mindfulness, both in formal and informal ways. Depending on time and space conditions, the benefits of the intervention and/or practice could be significant in work productivity and holistic employee well-being.

The study has several limitations. First, the study was conducted as cross-sectional research, the causal relationship between mindfulness practice, work engagement, and job performance could not be established. Future studies can use a longitudinal research design to examine the timing relationships of these variables. Second, this study controlled limited variables in the analyses, and may suffer from the unobserved-variable bias that may affect the estimates of mindfulness practice, job demands, and work engagement on job performance. Third, social workers have diverse work roles and clients that could produce an excessive effect on their response to job demands, mindfulness practice, and work engagement; thus, a future study on the diverse of social work field is needed. Fourth, the data collected on mindfulness practice, work engagement, and job performance were from self-reports that might have reporting errors. Mindfulness practice was assessed by two Likert-scale questions. Though this self-report measurement was consistent with the literature [48], future research could examine reliability and validity of this measurement and consider using other methods such as data triangulation through alternative reports. Finally, the research sample was exclusively from social workers in Guangzhou; it requires further investigation to test whether the finings can be applied to other social workers in China.

## 6. Conclusions

This study used 537 social workers in Guangzhou, China, to examine the effect of mindfulness practice on job performance and whether work engagement mediated the effect. The findings of this study provide evidence of a mediational pathway between mindfulness practice on job performance through work engagement. The findings contribute to the growing body of cross-cultural research that supports mindfulness practice as a resource that can promote work engagement, performance, and well-being of various occupational groups in the Chinese context. This study also provides evidence on the effects of both formal and informal mindfulness practice on work engagement and job performance in social workers in China. The results call for introducing mindfulness intervention and practice for Chinese social workers to advance their work engagement and job performance, which will have vital effects on the vulnerable populations they serve.

## Figures and Tables

**Table 1:** Descriptive statistics and correlations of key variables.

	Mean (SD)	1	2	3	4
1. Job Performance [0–6]	4.0 (0.8)	---			
2. Work Engagement [0–6]	3.5 (1.3)	0.59 ***	---		
3. Mindfulness Practice [0–6]	2.8 (1.5)	0.22 ***	0.34 ***	---	
4. Job Demands [1–7]	4.7 (0.7)	0.04	−0.17 ***	−0.04	---

Note: N = 537; *** *p* < 0.001. Numbers in brackets show ranges of the variables.

**Table 2:** Direct, Indirect, and Total Effects of SEM.

Path	Direct Effect	Indirect Effect	Total Effect
Specification 1: Mindfulness Practice			
Mindfulness Practice -> Work Engagement	0.33	---	0.33
Job Demands -> Work Engagement	−0.16	---	−0.16
Mindfulness Practice -> Job Performance	---	0.21	0.21
Job Demands -> Job Performance	0.15	−0.10	0.05
Work Engagement -> Job Performance	0.62	---	0.62
Specification 2: Formal Mindfulness Practice			
Formal Mindfulness Practice -> Work Engagement	0.21	---	0.21
Job Demands -> Work Engagement	−0.17	---	−0.17
Formal Mindfulness Practice -> Job Performance	---	0.13	0.13
Job Demands -> Job Performance	0.15	−0.10	0.05
Work Engagement -> Job Performance	0.62	---	0.62
Specification 3: Informal Mindfulness Practice			
Informal Mindfulness Practice -> Work Engagement	0.36	---	0.36
Job Demands -> Work Engagement	−0.15	---	−0.15
Informal Mindfulness Practice -> Job Performance	---	0.22	0.22
Job Demands -> Job Performance	0.15	−0.09	0.06
Work Engagement -> Job Performance	0.62	---	0.62

## Data Availability

Data available on request due to privacy restrictions.

## Appendix A

**Table A1:** Scale Items.

**Job Performance**
**Task Performance**
1. Achieves the objectives of the job
2. Meet criteria for performance
3. Demonstrates expertise in all job-related tasks
4. Fulfills all the requirements of the job
5. Could manage more responsibility than typically assigned
6. Appears suitable for a higher-level role
7. Is competent in all areas of the job, handles tasks with proficiency
8. Performs well in the overall job by carrying out tasks as expected
9. Plans and organizes to achieve objectives of the jobs and meet deadlines
**Contextual performance**
1. Helps other employees with their work when they have been absent
2. Volunteers to do things not formally required by the job
3. Takes initiative to orient new employees to the department even though not part of his/her job description
4. Helps others when their workload increases
5. Assists others with their duties
6. Makes innovative suggestions to improve the overall quality of the department
7. Willingly attends functions not required by the organization, but helps in its overall image
**Work Engagement**
1. At my work, I feel bursting with energy
2. At my job, I feel strong and vigorous
3. When I get up in the morning, I feel like going to work
4. I am enthusiastic about my job
5. My job inspires me
6. I am proud on the work that I do
7. I feel happy when I am working intensely
8. I am immersed in my work
9. I get carried away when I am working
**Job Demands**
1. Do you have too much work to do?
2. Do you have to work extra hard in order to complete something?
3. Do you have to hurry?
4. Would you prefer a calmer work pace?
5. Does your work demand a lot from you emotionally?
6. In your work, do you have to be able to convince or persuade people?
7. Are you confronted with things that affect you personally in your work?
8. Does your work put you in emotionally upsetting situations?
9. Have the proposed changes in your tasks been introduced well?
10. Do you find it difficult to adapt to changes in your tasks?
11. Do the changes in your tasks cause you problems?
12. Do the changes in your tasks have negative consequences for you?
